# A Plea for the Preservation of the Natural Teeth

**Published:** 1899-12

**Authors:** C. N. Johnson

**Affiliations:** Chicago, Ill.


ARTICLE IV.

### A PLEA FOR THE PRESERVATION OF THE NATURAL TEETH.

C. N. JOHNSON, L. D. S., D. D. S., CHICAGO, ILL.

It would appear to be the manifest duty of every man, in whatsoever station in life his lot be cast, to aim at the accomplishment of that which will result in the greatest possible good to the community in which he labors. More especially would this seem to be true in the case of those entrusted with the health and morals of their fellowman. A merchant may sell his customer a defective piece of goods and thereby stamp himself a dishonest man, but the real evil of such a procedure eventually falls upon the individual who perpetrates the wrong rather than upon the one who is wronged. A dealer in horses may misrepresent the soundness of an animal and complacently claim that it is merely one of the "tricks of the trade." Both the merchant and the horse dealer are offenders against the morals of the community, and are to that extent bad citizens, but the issue of their misdemeanors is more or less restricted and not in the broadest sense a serious menace to the welfare of the commonwealth.

Not so with the transgressor in certain other walks of life. The man who has in his keeping the physical well-being of the people cannot be lax in his methods without setting in motion a train of evils, the consequences of which may work havoc in generations yet to come. The duties of the professional man assume a more exalted bearing and hold a closer relationship with grave responsibilities than those engaged in the marts of trade.

These considerations are suggested in studying our present status of accomplishment in the practice of dentistry. Are we doing as much as we might do for the welfare of humanity? We are advancing rapidly in the mechanical perfection of our art, and are supposed to be a most progressive profession, but in the broader sense of a high moral purpose, are we living up to our greatest possibilities? In one respect at least I am constrained to believe that the true answer must be in the negative.

It will probably be admitted by all that the greatest possible service the dental profession can render humanity is to preserve the natural teeth in a state of health and usefulness. Is the profession doing this to the full extent of its capabilities? For answer I ask you to study the mouths of the people on our public thoroughfares or in an average assemblage of average citizens. In too many instances we see the mouth in its artificial environment marring the features of otherwise intelligent and beautiful individuals.

While this may not be directly construed as a reflection on the dental profession in all instances, yet if the matter be sifted to its legitimate source it will be found that at bottom the profession is most responsible for it.

A people are largely influenced by the trend of thought followed by their professional advisers. One of the main offices of a professional man is to educate the community to the highest and best of which his profession is capable, and as an evidence that this may be successfully accomplished, we see material differences in the existing state of the teeth in different communities. In some localities we find the mouths well cared for and the natural teeth for the most part reserved, while in others the teeth are allowed to run riot and give way to artificial substitutes without number. And it is just at this stage of the present paper that I wish to begin to particularize. My purpose in bringing this subject before the dentists of Ontario is fostered by the fact that within the range of my experience I see more artificial teeth according to population worn in Ontario than in any other place I have ever visited. Lest there may appear to be a shade of provincial prejudice in such a statement, I hasten to add that I was formerly a practitioner in this fair Province myself, and am free to acknowledge that in all probability while here I put in my full quota of ill advised and inharmonious artificial teeth. For this species of mild malpractice I am impelled even at this late date to offer my humble apologies to the people, and to the profession whose standard I to that extent degraded.

And yet, in my most magnanimous mood I cannot quite bring myself to admit that in those days there was anything like the slaughter of natural teeth that we see to-day. I can vividly recall my own early efforts to educate the people ih the care and preservation of the natural teeth, and while, as just admitted, I did not do all that I should have done in that direction, yet I feel that even in those days I accomplished something for the good of the people and the profession. It may be claimed that there was then a larger opportunity for doing good, with a smaller measure of discouragement than there is to day. Dental offices did not exist in departmental stores, and the day of the full-fledged five dollar-a-set man had not yet arrived. But the greater the necessity the more earnest should be the effort.

And this leads me logically into the consideration of some of the causes which have brought about the present status of dental practice in the Province. I have said that I see more artificial teeth worn here than anywhere else. I certainly see more young people with artificial teeth or defective natural ones than in any community I have ever visited. In encountering this wholesale exhibition of china-ware I have been led to look into the reasons for such a condition of things. Of course it may be stated on general principles that the prime factor in the case is a lack of knowledge on the part of the masses as to the real value of the natural organs, and the necessity for preserving them in a state of health. In the minds of the people there is not a distinction between the serviceability of a perfect set of natural teeth and a set made by the dentist. This lack of education is often attributed to a fundamental obtuseness on the part of the community, and I have noted that a great many dentists, when taken to task for allowing such a state of affairs, take refuge behind the assertion that it is impossible to educate their patrons up to a proper appreciation of the highest class of dental service.

It is probably true that certain communities are more difficult to educate than others, and that in some instances it is uphill work to attempt a reform. But I hold to the conviction that the duty of the dentist never stops short of an honest and persistent effort to enlighten the people who come under his care, no matter what their station in life. This effort must not consist merely in spasmodic and occasional dissertations presented in a half-hearted way, and lacking the force of conviction on their face. But it should be a living and abiding faith held sacred by the tenets of professional and humanitarian responsibility, and should constitute itself a feature of every-day practice to be pursued as conscientiously as any other part of office routine.

The dentist who has the welfare of his patients and his profession sincerely at heart will never deem it too much trouble to enter into explanations and offer painstaking advice to even his lowliest patron. His efforts may all too often fall on an unrequiting soil, and he may meet with countless rebuffs and discouragements, but in the end, with daily endeavor, he will see the happy results of his sowing, and if he does not succeed in leavening the whole lump, he will at least immeasurably raise the status of dental practice in his community.

There are many methods of interesting people in these matters, and the dentist must employ tact in his management of the different classes which come under his care. It will not suffice simply to make an unvarnished statement to the effect that the natural teeth are better than artificial ones. The lessons must be driven home by a quickwitted grasp of the situation, and an attack on any vulnerable point the patient may present. No two people can be managed precisely alike. As a practical illustration to rivet attention in certain cases, let me suggest a line of argument something like the following: Suppose your patient assumes—as patients sometimes are prone to do—that a set of artificial teeth answers every purpose, and that it is therefore not worth while striving to save the natural ones. If you ask that patient to admit that perfect mastication is neccessary to the most perfect health, he will usually agree with the proposition, because this is a generally recognized fact among all classes. The admission will likely be accompanied, however, with the statement that people are able to masticate with artificial teeth. Then is the time you have your patient at your mercy. You can go at him with cold facts in such array that he must retreat crestfallen from the controversy. Tell him that experimentation has shown, that when the natural teeth are in good condition the jaws are capable of closing with a force reaching in some instances to nearly, or quite, 300 pounds; that the average individual can exert more than 100 pounds pressure with the natural teeth; and that to properly masticate ordinary beefsteak it is necessary to use at least seventy or eighty pounds—to say nothing of the extraordinary article so often palmed off on a long-suffering public. Then ask him how many of his friends wearing artificial teeth are able to exert seventy pounds pressure with them? He will begin to take on a helpless look by this time, and you can clinch your argument by saying that the average force exerted by artificial teeth runs along about fifteen or twenty pounds. Tell him that artificial teeth would be smashed to smithereens as fast as they could be inserted if it were possible to use them as the natural teeth are used. Such a presentation of the subject will at least cause your patient some reflection, and start him thinking on a problem that never before entered his mind. I might also add that this form of reasoning may not be altogether lacking in moral features with a great many of our dental friends themselver.

The dentist owes it to his patients to study up ingenuous methods of argument to gain his point in their general enlightenment, and the man who devotes himself conscientiously to the better education of his patients will reap not only the reward of an appreciative *clientele*, but in the end will receive material recompense in a higher remuneration for his services.

But to return to the previous question. The universal use of artificial teeth must not altogether be laid at the door of ignorance. In latter years, when the stress of financial depression has pinched the people on every hand, the question of cost has had its influence. It has, in many cases, been cheaper to fill the mouth with porcelain than to preserve enemal and dentine. This is one of the abominations of low-grade dentistry. Not that it is a misfortune to have the poor man readily supplied with artificial substitutes at nominal cost when the natural teeth are irretrievably gone, but that the low price of artificial teeth has resulted in the sacrifice of innumerable natural teeth that would otherwise have been preserved. I have heard an eminent dentist make the statement, that it would have been better for the people and the profession, if artificial teeth had always been ten times the prevailing price.

But, after all these considerations are stated, I am strongly inclined to the belief that the greatest of all causes leading to the wholesale use of imported chinaware in the mouths of our patrons has yet to be mentioned. I may be treading on delicate ground, but I take the step boldly because I believe my impression to be correct. It is my conviction that much of the indifference manifested by the people in regard to saving the natural teeth is born of the fact, that failure has too often followed an honest attempt on their part to save them. In other words, our profession has not lived up to the highest possibilities of the science and art of dentistry as applied to preservation of the natural teeth. Patients have their teeth filled and pay for it, only to find in a few years—sometimes in a few months—sometimes even in a few weeks, the work undone, and the last condition of that mouth worse than the first. It requires not many experiences of this kind to foster the idea that teeth cannot be saved, and that the practice of filling them is a delusion and a snare. I have heard this argument used many and many a time by people who referred to their own experience as proof. This brings us squarely face to face with the question as to the probable permanency of fillings when properly inserted. Is it by virtue of necessity that so many fillings fail after a limited service? Must we acknowledge that with all our boasted handicraft we are able to accomplish nothing but the most temporary results in our operations? A few years ago a practitioner published a statement wherein he sought to prove by tabulated records, that the average duration of a gold filling was only about three years; and I very well remember a good friend of mine getting up in a meeting shortly after and claiming that he thought the estimate too high. I rebelled strenuously against such an assertion at the time, and, to-day, I rebel more than even, in the light of a careful study of my own records, after a continuous practice in one place of more than twelve years. I want to say to you gentlemen, in all sincerity, and with nothing but the most modest opinion of my own ability as an operator, that if I was not thoroughly convinced, that every day of my practice I was inserting fillings that would do service for ten, fifteen, twenty years—in fact, for the future lifetime of the patient, I should feel a sense of humiliation and defeat sufficient to stamp me in my own estimation as a failure among my fellow men. That this is no idle boast, and that I am not alone in the conviction, I give you the sentiments of two men of integrity in the profession, both modest and reliable, both carefully studious of their records, and each having practiced in his respective locality between thirty and forty years. One of them is now dead, the other living. One said to me, when questioned on the subject, after due deliberation and a modest estimate of his work—he was, in reality, one of the most modest men I ever knew—that, "with a conservative statement, he could claim for his gold fillings an average service of at least fifteen years." That may appear astounding, and even ridiculous to those who did not know this man, and who are viewing every day the work of the average operator, but I knew him well enough to be assured of his sincerity in making the statement, and I have seen sufficient of his work to convince me that he was not over sanguine in his estimate.

The other man, in discussing this subject with me on one occasion, made the assertion that, "with the exception of those occasional cases, where there seems to be an intensely active tendency to caries, that gold fillings inserted under favorable conditions, and with a full observance of the most approved principles, will last practically a lifetime.

Here we have in these two examples an inspiration toward the accomplishment of all that is greatest and best in our profession, and these men, with others of their ilk, have stamped the seal of professional stability on the records of the past, and pointed out the future possibilities of the highest class of dental service.

The chief difficulty with the average practitioner of dentististry is, that, in his daily work, he does not look carefully enough into the relation of cause and effect. He sees that a filling has failed in a tooth, but he does not stop to study the reason for that particular failure. He knows that, in one case a filling will do good service, where in another, with apparently equal care and similar conditions, his work seems to go for naught. His usual explanation is, that "in the one case, he is dealing with 'hard teeth,' and in the other, with 'soft teeth,'" but recent investigation has proved that there is little intrinsic difference in the structure of teeth of different individuals, and that even where there is a slight variation it seems to have little or no effect on the carious process. The fact is that we must cease hedging ourselves behind this story of "soft teeth," and must no longer offer it as an excuse for the failure of our operation. There are other causes at work which render it more difficult to save some teeth than others—causes which require careful study, but which cannot be considered in the present paper. Incidentally, however, it may be proper to state that the question is one of immunity from caries, or susceptibility to caries, much the same as we find immunity or susceptibility in other diseases. And, while on this subject, let me pause long enough to call attention to one feature in connection with it which seems to me to be of paramount interest, and to offer us the greatest possible encouragement in the management of those especially difficult cases, where the process of decay seems so rampant as to dishearten the most persistent and painstaking operator. Clinical experience goes to prove that in the vast majority of patients this intense susceptibility to caries is seldom constant. In other words we may find in one of our young patients the teeth breaking down at an alarming rate. Decay recurs around fillings in a discouraging manner, and new cavities crop out on all sides. Usually a case like this is given up as hopeless and the teeth sacrificed, but if the operator will only have the courage and patience to vigorously fight back the outbreak, he will find in nine cases out of ten, that, when least expected, there will be a change in the susceptibility, and the carious process will practically cease. I have seen this occur so often that I am no longer daunted when the worst possible case presents itself, and I am the more encouraged to go on and do the best I can for my patients, in view of the experience of an old and reliable practitioner, who recently stated to me, that in all his career he had not met a dozen cases where the carious process had been continuously persistent. We owe it to our patients to take these cases vigorously in hand and do the best for hem that the most advanced teaching will permit us.

But there is still another feature of this matter relating to the failure of fillings that I wish briefly to touch upon. I have said that the average practitioner does not study closely enough the relation of cause and effect when a filling fails. How many operators carefully consider why it is that a certain filling in a molar, or bicuspid, for instance, is forced out of place in a few months, when another filling anchored in precisely the same way remains secure for years? Did it ever occur to you that the force of mastication differs greatly in different individuals, and that in the one instance there was double the amount of pressure exerted on the filling to dislodge it that there was in the other? I have just stated that some individuals are able to close the jaws with a force of 300 pounds. Others, even with their natural teeth, can scarcely reach half that amount, while it has been learned that in the natural process of mastication there is the widest variation in the force exerted. Would any intelligent man expect to anchor a filling in a mouth where there was the maximum force in the same way that he would where there was the minimum force and expect it to remain equally well? And yet dentists every day are inserting fillings without the slightest reference to the stress which is likely to come upon them. I commend a study of this matter to the members of the Ontario Society, with the belief that they shall thereby proceed more intelligently in the anchorage of their fillings.

If dentists would develop definite modes of thought, and would carefully search out the causes of each failure that presents itself so as to avoid a repetition in the future, it would soon immeasureably increase their usefulness and add materially to the permanency of their operations. It would then not be long before the people would appreciate the benefits of dental science, and the hue and cry of to-day which seems to have for its watchword, "artificial teeth forever and everywhere," would give way to a proper respect for the natural organs, and would place dentistry in a more honorable light before the world.

I have written already too long, but, if time permitted, I should like to go more fully into the dètails of what I conceive to be a growing evil—the present defilement of the human face divine. What shall the dentist of the present have to answer for when, in a half century hence, the results of his mad havoc of to-day shall have left their indelible mark on the physiognomy of the nation? We are making for good or ill in all we do, not only fcr the present, but for future generations; and it is meet for us, that we so discharge our bounden duty, that posterity, instead of heaping maledictions on us for the disfigurement we have wrought, may find it in their heart of hearts to rise up and call us blessed.—*Dominion Dental Journal.*

